# National health insurance subscription and maternal healthcare utilisation across mothers’ wealth status in Ghana

**DOI:** 10.1186/s13561-017-0152-8

**Published:** 2017-04-26

**Authors:** Edward Kwabena Ameyaw, Raymond Elikplim Kofinti, Francis Appiah

**Affiliations:** 1 Department of Population and Health, Faculty of Social Sciences, University of Cape Coast, Cape Coast, Ghana; 2 Department of Economics, Faculty of Social Sciences, University of Cape Coast, Cape Coast, Ghana

**Keywords:** Antenatal care, Maternal healthcare utilisation, Wealth status, Women, Health insurance

## Abstract

**Introduction:**

This study is against the backdrop that despite the forty-nine percent decline in Maternal Mortality Rate in Ghana, the situation still remains high averaging 319 per 100,000 live births between 2011 and 2015.

**Objective:**

To examine the relationship between National Health Insurance and maternal healthcare utilisation across three main wealth quintiles (Poor, Middle and Rich).

**Methods:**

The study employed data from the 2014 Ghana Demographic and Health Survey. Both descriptive analysis and binary logistic regression were conducted.

**Results:**

Descriptively, rich women had high antenatal attendance and health facility deliveries represented by 96.5% and 95.6% respectively. However, the binary logistic regression results revealed that poor women owning NHIS are 7% (CI = 1.76–2.87) more likely to make at least four antenatal care visits compared to women in the middle wealth quintile (5%, CI = 2.12–4.76) and rich women (2%, CI = 1.14–4.14). Similarly, poor women who owned the NHIS are 14% (CI = 1.42–2.13) likely to deliver in health facility than women in the middle and rich wealth quintile.

**Conclusion:**

The study has vindicated the claim that NHIS Scheme is pro-poor in Ghana. The Ministry of Health should target women in the rural area to be enrolled on the NHIS to improve maternal healthcare utilisation since poverty is principally a rural phenomenon in Ghana.

## Background

Maternal related complications constitute the major source of disabilities and mortality among women within reproductive age globally [1]. Despite the tremendous progress made by the global community in combating maternal related complications and mortality, 289,000 women still die yearly owing to pregnancy with low and middle income countries bearing the highest brunt [1]. The disparity between these countries and the developed countries presupposes that income disparities have consequences on maternal health status. The crucial nature of maternal and child health instigated the global community to devote the third Sustainable Development Goal (SDG) to reduction in maternal mortality, neonatal, infant and under five mortality rates [2].

Whilst studies indicate decline in maternal mortality rates since 1990s, the decline is not universal and still remains high in southern Asia and Africa [1, 3, 4]. For instance, it has been realised that risk of maternal mortality for a woman in Sub-Saharan Africa is forty-seven times higher as compared to someone in the United States, meanwhile most of these deaths are avoidable [5]. In the case of Ghana, despite the forty-nine percent decline in Maternal Mortality Rate (MMR), the situation still remains high because as noted by the World Bank, it averaged 319 per 100,000 live births between 2011 and 2015 [6].

The high rate of maternal complications emerge from the numerous threats confronting the health sector of most African countries and cost associated with healthcare utilisation. Arguably, economic standing of women is critical to the extent to which maternal healthcare can be utilised and as indicated by Marmot [7], income relates to health in three principal ways: countries’ gross national product; individual’s income; and variation in income. In light of this, the poor are the most vulnerable in terms of maternal healthcare access [8, 9]. Since investment in maternal health constitutes not only social and political imperatives but also cost effective investment, a number of initiatives have been instituted manifesting in interventions such as health insurance scheme.

Health insurance exists as an essential pro-poor initiative and despite its enormous benefits, evidence suggests that maternal healthcare utilisation especially Antenatal Care (ANC) attendance and supervised delivery are still induced by maternal wealth status in some countries [10]. Through rise in health insurance subscription, a growing body of studies have investigated the essence of health insurance to utilisation of healthcare [11–14], meanwhile, the impact of health insurance on maternal healthcare utilisation across wealth status in Ghana has not gained recognition in literature.

The National Health Insurance Scheme (NHIS) commenced in 2005 as a demand side initiative to overcome financial obstacles to healthcare utilisation. As far as the link between NHIS ownership and maternal healthcare utilisation is concerned, some questions remain unanswered in the literature: (1) is NHIS a prerogative of the rich, the poor or both?; (2) which of these groups should be the primary target of the NHIS?; and (3) which of these groups are currently benefitting from NHIS ownership via maternal health utilisation? Whilst some evidence point out that women with higher socio-economic standing least utilise NHIS due to their access to multiple options to enhance their health status in terms of accessing quality and or private healthcare and good nutrition during pregnancy [15, 16], counter evidence have also been reported [17]. Considering the fact that divergent results have been reported in Ghana about NHIS and maternal healthcare utilisation [18, 19], this study intends to unearth the current direction as far as the relationship between health insurance and maternal healthcare utilisation across wealth status is concerned. There is therefore the need for this investigation with the 2014 Ghana Demographic Health Surveys (GHDS) to know the current status of how well the NHIS has impacted maternal healthcare utilisation (antenatal visits and place of delivery) across wealth status in Ghana.

### Theoretical framework

Several and complex drivers influencing healthcare utilisation revolve around social, cultural, economic and religious factors [20, 21]. In order for the concept of healthcare utilisation and its drivers which are at the core of this paper to be well-understood and for conceptual clarity, it demands theoretical guidance. Anderson’s Behavioural Model (BM) of healthcare utilisation [22, 23] shall guide this paper since the primary focus of the paper is to investigate the relationship between insurance subscription and maternal healthcare utilisation (measured by ANC attendance and place of delivery) across wealth status of women (poor, middle, rich).

The BM postulates that healthcare utilisation rest on predisposing, enabling and need factors operating at both individual and contextual domains [23, 24]. Within the predisposing factors, individual predisposing factors constitute the aggregate of demographic, biological and social while the contextual predisposing factors also encompass demographic, social composition of communities and cultural norms that interact to influence healthcare utilisation [23, 24]. The enabling factors include but not limited to one’s income and wealth status that enables individuals to pay for healthcare services and the effective price of healthcare influenced by one’s health insurance status. However, due to inherent weaknesses, the model was revised and as such healthcare systems, service availability, population-based factors and consumer satisfaction in the initial model are considered as drivers of healthcare utilisation.

## Methods

Data from the 2014 GDHS was used for this study. Specifically, the women and child files were used for the study. GDHS is carried out by the Ghana Statistical Service and Macro International under the auspices of DHS programs. The survey captures data on various aspects of maternal health conditions within the country and as such was deemed suitable for this study. The dataset was requested online from Measure DHS website on the 16th October, 2015. In all, 9,396 women (aged 15–49) from 11,835 households nationwide were interviewed [25]. However, 4,294 women had birth history within the last 5 years preceding the survey and as such they constituted the sample size for this study.

The 2014 GDHS was conducted with an updated frame from the 2010 Population and Housing Census (PHC) prepared by the Ghana Statistical Service (GSS). The frame exempted institutional and nomadic groups including hotel occupants and prisoners. The survey constituted a two-stage sample design for the purpose of allowing estimates of core indicators at the national level. The initial phase constituted selection of sample points (clusters) involving enumeration areas (EAs) outlined for the 2010 PHC in which 427 clusters were designated in all constituting 216 from urban and 211 from rural areas. The next stage utilised systematic sampling of households in which household inventory operation was carried out in all the identified EAs between January and March 2014. Afterwards, the households to be considered for the survey were selected from the list randomly [25].

### Econometric model

To investigate the effect of National Health Insurance Subscription (NHIS) on maternal healthcare utilisation across the three main wealth quintiles (Poor, Middle and Rich) in Ghana we relied on theorising maternal healthcare utilisation specified by Anderson [22, 23]. The study assumes that the mother derives utility from (1) making at least four antenatal care visits during pregnancy and, (2) delivering in a health facility/hospital, and that there is disutility to the mother and the husband in the form of complications during pregnancy and time of delivery when the mother fails to either make at least four antenatal care (ANC) visits or delivers at a health facility. It can be elaborated that the mother makes a conscious effort to improve her own survival and that of the child during pregnancy through investment in health care which can take the form of either curative or preventive health care. Therefore, it can be argued that the decision of the mother to utilise maternal health is the responsibility of the mother as Anderson [22] later considered individuals as the unit of analysis which goes beyond health care utilisation only.

The probability that the mother utilises maternal health care is a function of a key enabling factor of National Health Insurance Subscription NHIS **(** ***N*** **)** and the level of education of the mother **(** ***E***_***m***_ **),** the level of education of the partner/husband **(** ***E***_***f***_ **)**. Some of the predisposing factors considered for the study are religious affiliation **(** ***R***_***A***_ **)**, household purchasing decision **(** ***HP*** **)** and household health care decision making (***HC***). The maternal health care probability production function of the mother is thus specified as:

1 $$ \boldsymbol{M}{\boldsymbol{H}}_{\boldsymbol{i}} = \boldsymbol{\pi} \left(\boldsymbol{N},{\boldsymbol{E}}_{\boldsymbol{m}},{\boldsymbol{E}}_{\boldsymbol{f}},{\boldsymbol{R}}_{\boldsymbol{A}},\ \boldsymbol{HP},\ \boldsymbol{HC},\ \boldsymbol{X}\right) $$

Where *MH*_*i*_ is the probability that the mother utilises the two maternal health care services: (1) probability that the mother makes at least four antenatal care visits; and (2) the probability that the woman delivers in a health facility, and ***X*** is a vector of other exogenous variables such as ecological zone and the urban dummy.

2 $$ \boldsymbol{M}{\boldsymbol{H}}_{\boldsymbol{i}} = {\boldsymbol{\varphi}}_{\boldsymbol{i}}\boldsymbol{\beta} + {\boldsymbol{\delta}}_{\boldsymbol{i}}\;\mathbf{with}\kern0.36em \boldsymbol{M}{\boldsymbol{H}}_{\boldsymbol{i}} = \Big\{{}_{0\ \boldsymbol{otherwise}}^{1\ \boldsymbol{if}\ \boldsymbol{M}{\boldsymbol{H}}_{\boldsymbol{i}} > 0} $$

Where *MH*_*i*_ is the probability of maternal health care utilisation by the mother, which is broken up into two in this study: (1) the probability that the mother makes at least four antenatal care visits; and (2) the probability that the mother delivers in a health facility. Equation two is therefore estimated for the three wealth quintiles, viz., poor, middle and rich. ***φ***_***i***_ is a vector of exogenous factors influencing maternal health care utilisation; ***β*** is a vector of unknown parameters; and *δ*_*i*_ is an error term with zero mean and a constant variance, which also captures the unobserved factors in the model. In order to estimate equation (2), the maximum likely estimation (MLE) technique in logistic regression is employed. This is against the background that the logistic regression satisfies the main assumption underlying MLE, viz., the dependent variable, maternal health care utilisation, is dichotomous.

### Dependent variables

Utilisation of maternal healthcare services, comprising ANC attendance and place of delivery, was the outcome variable. The first dependent variable, ANC visits was recoded into a binary outcome variable with zero ‘0’ denoting less than four and one ‘1’ at least four. Similarly, the second dependent variable, place of delivery, was recoded into a binary outcome variable with zero ‘0’ denoting delivery at home and one ‘1’ denoting delivery in a health facility.

### Independent variables of interest

The main independent variable of the study was health insurance ownership which is a dummy variable where one ‘1’ represents women who have subscribed to NHIS, and zero ‘0’ otherwise. The effect of NHIS ownership on maternal healthcare utilisation was analysed across the wealth status of women. Wealth status is a categorical variable with zero ‘0’ denoting women in the poor wealth quintile, one ‘1’ denoting women in the middle wealth quintile and two ‘2’ denoting women in the rich wealth quintile. As with any good model specification and taking into cognizance the theoretical literature review, other socio-demographic variables were controlled for in the estimation. These are residential status, religion, marital status, frequency of watching television and listening to radio, frequency of reading newspaper, ecological zone (made up of the ten administrative regions), occupation, partner’s occupation and education, contraceptive usage and birth order.

## Results

### Descriptive statistics for the independent variables

Table 1 presents the descriptive statistics of the independent variables. The analysis indicated that across wealth status, the highest NHIS subscription occurred among the poor (42.9%) whilst the least subscription occurred among those in the middle wealth status (19.4%). Specifically, 67.1% of the poor had subscribed to the scheme, whereas 68.7% of those in the middle wealth category had subscribed. However, among the rich women, NHIS subscription stood at 72.9%. It was found that most poor women reside in rural settings (77.1%) as compared to women in other wealth categories. Within the poor, rural residents accounted for 86.3%. This observation implies that most poor women in Ghana reside in rural settings. This residential status might have potential implications on their access to maternal health services. Poor women affiliated to non-Christian religious bodies (65.6%) exceeded their non-Christian counterparts in middle (16.0%) and rich (18.4%) wealth categories. The proportion of poor women who were Christians (46.9%) was the highest.

**Table 1:** Descriptive Statistics for the Independent Variables

Variable	Poor	Middle	Rich	*N* = 4,294
	Row (Col.)	Row (Col.)	Row (Col.)	
*NHIS*				
Owned	42.9 (67.1)	19.4 (68.7)	37.7 (72.9)	100
	45.0 (32.9)	21.8 (31.3)	33.2 (27.1)	100
Not Owned	(100)	(100)	(100)	
*Residence*				
Rural	77.1 (86.3)	16.7 (51.9)	6.2 (12.6)	100
Urban	17.2 (13.7)	21.8 (48.1)	61.0 (87.4)	100
	(100)	(100)	(100)	
*Religion*				
Others	65.6 (35.6)	16.0 (24.0)	18.4 (18.0)	100
Christianity	46.9 (64.4)	20.0 (76.0)	33.1 (82.0)	100
	(100)	(100)	(100)	
*Marital Status*				
Not Married	48.6 (32.1)	26.2 (48)	25.2 (30.1)	100
Married	54.1 (67.9)	15.0 (52)	30.9 (69.9)	100
	(100)	(100)	(100)	
*Occupation*				
Not working	45.4 (15.0)	24.1 (22.2)	30.5 (18.2)	100
Working	53.7 (85.0)	17.7 (77.8)	28.6 (81.8)	100
	(100)	(100)	(100)	
*Partner’s occupation*				
Primary	87.5 (9.6)	9.6 (20.9)	2.9 (45.9)	100
Secondary	23.9 (76.3)	30.7 (25.0)	45.4 (4.6)	100
Tertiary	23.1 (14.1)	16.9 (54.1)	60.0 (49.5)	100
	(100)	(100)	(100)	
*Education*				
No education	80.6 (51.4)	12.0 (21.2)	7.4 (8.5)	100
Primary	61.6 (23.9)	20.3 (21.8)	18.1 (12.7)	100
At least secondary	27.8 (24.7)	23.1 (56.9)	49.1 (78.9)	100
	(100)	(100)	(100)	
*Partner’s education*				
No education	85.9 (48.4)	8.5 (14.2)	5.5 (5.6)	100
Primary	38.8 (50.3)	21.7 (83.0)	39.5 (93.1)	100
At least secondary	44.1 (1.3)	32.2 (2.8)	23.7 (1.3)	100
	(100)	(100)	(100)	
*Ecological zone*				
Coastal	27.1 (14.7)	23.7 (35.6)	49.2 (48.1)	100
Savannah	81.2 (33.6)	8.3 (49.9)	10.5 (39.8)	100
Forest	45.6 (51.7)	24.4 (14.5)	30.0 (12.1)	100
	(100)	(100)	(100)	
*Frequency of listening to radio*				
Not at all	69.8 (25.7)	14.9 (15.2)	15.3 (10.1)	100
Less than once a Week	50.1 (29.4)	21.8 (35.5)	28.1 (29.8)	100
At least once a week	46.7 (44.9)	18.6 (49.4)	34.7 (60.1)	100
	(100)	(100)	(100)	
*Water source*				
Pipe	5.3 (1.0)	13.3 (5.1)	81.4 (20.2)	100
Others	48.0 (99.0)	21.0 (94.9)	31.0 (79.8)	100
	(100)	(100)	(100)	
*Contraceptive usage*				
No modern contraceptive	43.3 (75.5)	19.8 (71.7)	36.9 (73.9)	100
Uses modern	44.6 (24.5)	22.1 (28.3)	33.3 (26.1)	100
Contraceptive	(100)	(100)	(100)	
*Healthcare decision making*				
Alone	46.3 (19.0)	20.3 (25.3)	33.4 (24.4)	100
Not alone	54.7 (81.0)	16.7 (74.7)	28.6 (75.6)	100
	(100)	(100)	(100)	
*Household purchase decision making*				
Alone	50.7 (17.9)	19.6 (20.9)	29.7 (18.7)	100
Not alone	53.4 (82.1)	16.9 (79.1)	29.7 (81.3)	100
	(100)	(100)	(100)	
*Decision making on visits*				
Alone	48.9 (20.2)	18.9 (23.7)	32.0 (23.5)	100
Not alone	53.9 (79.8)	17.0 (76.3)	29.0 (76.5)	100
	(100)	(100)	(100)	
*Shared toilet facility*				
No	1.7 (0.6)	8.4 (5.9)	90.0 (58.3)	100
With other household only	53.2 (99.4)	22.9 (94.1)	23.9 (41.7)	100
	(100)	(100)	(100)	

Computed from GHDS 2014 Data

It was found that across wealth status, marriage was high among poor women (54.1%). Similarly, marriage stood at 69.9% among the rich women. With regard to occupation across wealth status, it was realised that the greatest proportion of working women were poor (53.7%) with the least being women in the middle wealth status (17.7%). As depicted in Table 1, most women whose partners were engaging in primary occupation were poor (87.5%) when considered across wealth status. On education, most uneducated women were poor (80.6%) whereas the highest proportion of those with at least secondary education was recorded among rich women (49.1%). Specifically, 51.4% of the poor were uneducated with 24.7% having at least secondary education. Also, majority of the rich women had at least secondary education (78.9%) whilst only 8.5% had no formal education. This finding imply that at least two out of ten Ghanaian women in the reproductive age group have had some formal education.

Upon analyzing partners’ education, it was noted that partners of most poor women were uneducated (85.9%). Investigation among the poor indicated that half of their partners had attained primary education (50.3%) as indicated in Table 1. Similarly, majority of the rich women’s partners had attained primary education (93.1%). Although educational attainment is generally high among the women, it is obvious that women from wealthier homes have dominated. It was evident from the study that a significant share of the rich women were within the Coastal zone (49.2%) with major of the poor residing in the Savanna zone (81.2%). Further analysis indicated half of the poor women were within the Forest zone (51.7%).

Most poor women were found not to listen to radio at all (69.8%) as compared with women in other wealth categories. Among the poor, 44.6% were listening to radio at least once a week with 25.7% not listening at all. With respect to the rich, 60.1% were listening to radio at least once a week whilst 10.1% were not listening to radio. Upon exploring among women in these wealth categories, it was noted that almost all poor women obtained water from sources other than pipe (99.0%). Among those in the middle wealth status, 94.9% were obtaining water from other sources. Hence, a greater proportion of Ghanaian women obtain water from sources other than pipe. As such, it is more probable that sources such as bole holes, wells and streams are much utilised, meanwhile, the health implications of these sources are sometimes adverse.

Investigation into contraceptive use revealed that as compared to women in other wealth categories, non-use was high among poor women (43.3%). Specifically, 75.5% of the poor were not using contraceptives, meanwhile the proportion of the rich who were not using contraceptives stood at 73.9%. Analysis of decision making on healthcare unraveled that women who were not taking the decision alone were predominantly poor (54.7%) as compared with middle and rich women. Among the poor, 81.0% were not taking the decision alone, whereas 74.7% of those in the middle wealth status were also not taking the decision alone. Almost all poor women were sharing (99.4%) and this was not so different from the observation made among those in the middle wealth status as 94.1% were sharing toilet facility with other households.

### Maternal healthcare utilisation by wealth status

**Fig. 1:**
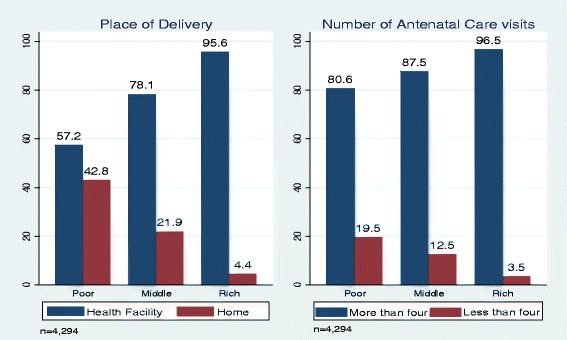
Maternal healthcare utilisation by wealth status. Source: GDHS 2014

Assessment of delivery in health facility across wealth status revealed that generally the rich tend to deliver in health facilities more than their poor counterparts. This is because 95.6% of rich women delivered in health facilities compared to 57.2% health facility deliveries among poor women as illustrated in Fig. 1. Similarly, attendance of antenatal care was relatively high among rich women (96.5%) than their poor counterparts (80.6%). The Figure has indicated a trend whereby the rich appears to make more use of maternal healthcare services (place of delivery and antenatal visits). The low utilisation among the poor do not necessarily indicate that they are not interested in accessing the services but might be disadvantaged by their low economic status. This is because the rich are more likely to have multiple avenues of accessing these services which the poor might not be able to utilise. From the Figure, more than half of poor mothers deliver at home (43%) compared to the mothers in the middle wealth quintile (22%) and rich mothers (4%). Similarly, majority of mothers who fail to make the recommended WHO healthy antenatal visits of four are poor, thus 19.9% compared to mothers in the middle and rich wealth quintile of 12.5 and 3.5% respectively.

### Maternal healthcare utilisation by wealth and zonal distribution

It was observed that home deliveries dominated among poor women in all the three zones of the country namely Coastal Poor (43.6%), Forest Poor (38.9%) and Savannah Poor (45.1%). At the same time, poor women in rural settings were noted to have the highest prevalence of home deliveries (45.2%) as depicted in Table 2. It is not surprising that rural poor women have high prevalence of home deliveries considering the poor road networks linking these rural areas to health facilities coupled with refusal of healthcare providers to accept postings to rural settings. When ANC visit was viewed across Rural–urban dimension, it was clear that having at least four visits was prevalent among both Rural Rich (97.4%) and Urban Rich (96.4%) as projected in Table 2.

**Table 2:** Maternal Healthcare Utilisation by Wealth and Zonal Distribution

	Place of delivery			ANC visit		
	Home	Health facility	Total	˂4	≥4	Total
Coastal Poor	43.6	56.4	100	17.9	82.1	100
Coastal Middle	29.0	70.9	100	11.9	88.1	100
Coastal Rich	5.7	94.3	100	3.2	96.8	100
Forest Poor	38.9	61.0	100	21.2	78.8	100
Forest Middle	18.4	81.6	100	13.6	86.4	100
Forest Rich	3.1	96.9	100	4.1	95.9	100
Savannah Poor	45.1	54.9	100	18.8	81.2	100
Savannah Middle	16.2	83.8	100	10.3	89.7	100
Savannah Rich	3.3	96.7	100	2.7	97.4	100
Rural Poor	45.2	54.8	100	20.4	79.6	100
Rural Middle	28.3	71.7	100	10.8	89.2	100
Rural Rich	9.1	90.9	100	2.6	97.4	100
Urban Poor	27.9	72.1	100	13.7	86.3	100
Urban Middle	14.8	85.2	100	14.4	85.6	100
Urban Rich	3.7	96.3	100	3.6	96.4	100

Computed from GHDS 2014 Data

### Results of econometric models (logistic regression)

#### Logistic regression results on ANC visit

In all, six logistic regression models were constructed in explaining the effect of health insurance ownership on maternal healthcare utilisation across wealth status. Table 3 presents the results of antenatal care visit whilst Table 4 presents the results of place of delivery. With regard to antenatal care (ANC) visits, the logistic regression analysis indicated that poor women who were subscribed to the NHIS were about 7% (CI = 1.76–2.87) likely to have more ANC visits than poor women who have not subscribed to the scheme. Whilst urban poor residents were about 3% (CI = 1.66–3.21) more probable to attend ANC than rural residents, poor Christians were about 5% less likely to attend ANC (−4.5%, CI = 0.67–1.34) as compared to poor women affiliated to other religions. Married women in the poor category were noted to have about 7% (CI = 1.41–2.44) likelihood of accessing ANC than the unmarried. Meanwhile, poor women who were working were 10% (CI = 1.11–2.26) more likely to access ANC than their non-working counterparts (reference category). Common knowledge would argue that non-working women might have had enough time to attend ANC as compared to working women but that is not the case in the Ghanaian context. This, however, points to the notion that attendance or non-attendance of ANC is not only a function of availability of time but perception about the need to access such service.

**Table 3:** Logistic regression results on ANC visit

			ANC			
Variable	Poor	95% CI	Middle	95% CI	Rich	95% CI
*Health Insurance*						
Not-subscribed		1,1		1,1		1,1
Subscribed	0.067**(0.028)	1.76–2.87	0.045 (0.031)	2.12–4.76	0.017 (0.013)	1.14–4.14
*Residence*						
Rural		1,1		1,1		1,1
Urban	0.026 (0.039)	1.10–2.68	−0.034 (0.026)	0.98–1.64	−0.012 (0.015)	0.28–2.73
*Religion*						
Others		1,1		1,1		1,1
Christianity	−0.045 (0.028)	0.67–1.34	0.034 (0.034)	0.46–2.51	0.005 (0.022)	0.59–4.21
*Marital Status*						
Not Married		1,1		1,1		1,1
Married	0.067*(0.031)	1.41–2.44	0.080**(0.031)	0.74–2.86	0.021 (0.016)	0.75–3.16
*Occupation*						
Not working		1,1		1,1		
Working	0.104**(0.044)	1.11–2.26	0.095**(0.036)	1.75–4.31	0.049**(0.018)	
*Partner’s occupation*						
Primary		1,1		1,1		1,1
Secondary	0.048 (0.032)	1.15–2.65	−0.002 (0.031)	2.31–3.99	0.010 (0.027)	0.23–2.30
Tertiary	0.129***(0.029)	1.27–3.87	0.027 (0.036)	1.54–2.56	0.019 (0.025)	0.65–5.42
*Education*						
No education		1,1		1,1		1,1
Primary	−0.009 (0.035)	0.86–1.69	0.012 (0.043)	0.77–1.75	−0.030 (0.025)	0.63–2.71
At least secondary	−0.001 (0.037)	0.91–3.01	0.049 (0.040)	0.94–1.88	0.005 (0.017)	0.97–1.72
*Partner’s education*						
No education		1,1		1,1		1,1
Primary	−0.019 (0.040)	0.80–1.64	−0.029 (0.049)	0.82–1.66	0.040 (0.035)	1.21–3.22
At least secondary	0.023 (0.036)	0.98–1.96	−0.007 (0.039)	0.78–1.84	0.041 (0.033)	2.12–4.21
*Ecological zone*						
Coastal		1,1		1,1		1,1
Savannah	0.015 (0.039)	0.58–1.37	0.015 (0.039)	0.49–1.52	0.001 (0.022)	0.82–2.45
Forest	−0.033 (0.030)	0.46–1.02	−0.033 (0.030)	0.64–1.73	−0.005 (0.012)	0.94–1.63
*Frequency of listening to radio*						
Not at all		1,1		1,1		1,1
Less than once a Week	0.042 (0.041)	1.00–1.84	0.042 (0.041)	2.02–4.89	−0.013 (0.017)	2.42–5.21
At least once a week	0.106***(0.036)	1.83–3.28	0.106**(0.036)	1.86–3.72	0.009 (0.012)	2.01–4.21
*Water source*						
Pipe		1,1		1,1		1,1
Others	0.281 (0.154)	0.45–2.14	0.089**(0.033)	0.65–2.71	0.009 (0.015)	0.72–2.71
*Contraceptive usage*						
No modern contraceptive		1,1		1,1		1,1
Uses modern Contraceptive	0.064**(0.025)	1.43–2.70	0.065**(0.024)	0.95–2.63	0.000**(0.012)	0.52–2.61
Birth order	−0.004*(0.032)	0.33–2.01	0.001 (0.154)	1.22–3.28	0.024 (0.031)	0.74–3.82
*Healthcare decision making*						
Alone		1,1		1,1		1,1
Not alone	−0.050 (0.028)	1.21–3.43	−0.052 (0.030)	1.63–3.21	−0.015 (0.011)	1.82–2.73
*Household purchase decision making*						
Alone		1,1		1,1		1,1
Not alone	0.080 (0.042)	2.32–4.51	0.070 (0.041)	2.41–4.22	−0.016 (−0.013)	0.54–2.82
*Decision making on visits*						
Alone		1,1		1,1		1,1
Not alone	0.021 (0.036)	1.32–4.12	0.075*(0.038)	0.56–2.61	0.004 (0.016)	0.82–2.64
*Shared toilet facility*						
No		1,1		1,1		1,1
With other household only	−0.026 (0.036)	2.11–4.24	−0.066 (0.053)	2.31–4.46	−0.026*(0.012)	2.14–4.62
Public	−0.004 (0.032)	0.97–2.54	−0.051 (0.049)	1.11–3.01	−0.013 (0.012)	1.85–3.22
Hosmer and Lemeshow’s Gof	9		12.7		9.7	

Co-efficient; standard error in bracket; 95% confidence interval *p < 0.10; **p < 0.05; ***p < 0.001

**Table 4:** Logistic regression on place of delivery

Variable	Poor	95% CI	Middle	95% CI	Rich	95% CI
*Health Insurance*						
Not-subscribed		1,1		1,1		1,1
Subscribed	0.139***(0.037)	1.42–2.13	0.074*(0.039)	1.00–2.10	0.048*(0.017)	1.02–3.18
*Residence*						
Rural		1,1		1,1		1,1
Urban	0.033 (0.053)	1.66–3.21	0.168***(0.035)	1.63–3.44	0.041 (0.022)	1.60–5.92
*Religion*						
Others		1,1		1,1		1,1
Christianity	0.087*(0.042)	1.16–1.78	0.076 (0.047)	0.75–1.80	0.029 (0.021)	0.71–2.97
*Marital Status*						
Not Married		1,1		1,1		1,1
Married	0.043 (0.040)	1.08–1.71	0.044 (0.038)	0.70–1.44	−0.001 (0.015)	0.70–2.96
*Highest Education*						
No education		1,1		1,1		1,1
Primary	0.075 (0.469)	1.09–1.84	0.024 (0.058)	0.98–1.15	−0.041 (0.024)	0.82–3.01
At least Secondary Education	0.171***(0.477)	1.53–2.80	0.094 (0.056)	1.01–2.75	0.011 (0.019)	1.12–2.84
*Partner’s Education*						
No education		1,1		1,1		1,1
Primary	−0.067 (0.051)	1.06–1.87	0.108 (0.078)	2.13–3.08	0.043 (0.039)	1.88–2.76
At least secondary Education	−0.018 (0.045)	0.93–1.60	0.094 (0.068)	1.75–2.37	0.053 (0.034)	2.71–3.98
*Frequency of listening to radio*						
Not at all		1,1		1,1		1,1
Less than once a week	−0.019 (0.050)	0.98–1.66	−0.065 (0.054)	2.07–3.55	−0.007 (0.021)	0.63–2.75
At least once a week	0.102**(0.045)	1.40–2.26	−0.041 (0.052)	1.89–2.33	0.020 (0.018)	2.71–4.87
*Ecological Zone*						
Coastal		1,1		1,1		1,1
Savannah	0.086 (0.053)	0.85–1.65	−0.015 (0.071)	0.82–1.99	0.026 (0.023)	1.53–3.44
Forest	0.049 (0.043)	0.73–1.39	0.017 (0.040)	2.14–3.11	0.027*(0.013)	2.31–5.44
*Occupation*						
Not working		1,1		1,1		1,1
Working	0.047 (0.049)	0.90–1.60	0.070 (0.049)	1.78–2.56	0.021 (0.018)	2.55–4.21
*Partner’s occupation*						
Primary		1,1		1,1		1,1
Secondary	0.048 (0.048)	0.86–1.60	−0.034 (0.043)	2.76–3.44	0.072 (0.040)	0.74–2.55
Tertiary	0.073 (0.056)	1.42–3.09	0.020 (0.057)	1.99–3.76	0.068 (0.041)	2.11–4.31
*Water source*						
Pipe		1,1		1,1		1,1
Others	−0.001 (0.119)	1.93–2.01	−0.081 (0.082)	2.12–3.71	−0.020 (0.014)	0.98–3.11
*Contraceptive usage*						
No modern contraceptive		1,1		1,1		1,1
Uses modern Contraceptive	0.022 (0.036)	1.18–1.87	0.009 (0.039)	1.53–2.76	−0.017 (0.015)	1.77–3.21
Birth order	0.018 (0.028)	0.78–1.98	0.025 (0.087)	2.33–3.81	0.038 (0.051)	0.84–3.22
*Healthcare decision making*						
Alone		1,1		1,1		1,1
Not alone	0.025 (0.468)	2.54–2.98	−0.049 (0.047)	0.63–1.21	−0.016 (0.015)	2.01–4.72
*Household purchase decision making*						
Alone		1,1		1,1		1,1
Not alone	−0.023 (0.048)	1.53–2.07	0.087 (0.057)	0.74–1.52	0.015 (0.021)	0.82–2.60
*Decision making on visits*						
Alone		1,1		1,1		1,1
Not alone	0.004 (0.049)	1.32–1.96	−0.014 (0.053)	0.69–2.11	0.001 (0.015)	2.03–4.82
*Shared toilet facility*						
No		1,1		1,1		1,1
With other household only	0.010 (0.048)	0.77–1.01	0.038 (0.070)	0.52–1.74	−0.019 (0.015)	0.93–3.11
Public	0.045 (0.044)	0.98–1.23	0.013 (0.067)	1.55–3.02	−0.032 (0.016)	1.71–3.85
Hosmer and Lemeshow’s Gof	9.9		13.5		7.3	

Co-efficient; standard error in bracket; 95% confidence interval *p < 0.10; **p < 0.05; ***p < 0.001

Poor women with primary education (−1.0%, CI = 0.86–1.69) and those with at least secondary education were all less probable to utilise ANC as compared to those without formal education, meanwhile, contrary observation was made among those whose partners had at least secondary education (2.3%, CI = 0.98–1.96). It was also evident that ANC visits among poor women listening to radio at least once a week was 10% (CI = 1.83–3.28) higher than those who did not listen to radio at all. Less possibility of ANC attendance was associated with poor women who could not decide on their healthcare alone (−5%, CI = 1.21–3.43) as compared to those taking such decision on their own. However, poor women who were unable to decide household purchases (8%, CI = 2.32–4.51) and visits (2%, CI = 1.32–4.12) alone were more probable to utilise ANC as compared to those taking such decisions alone.

Among those in the middle wealth status, higher likelihood of ANC visits was observed among those subscribed to the NHIS as compared to those who had not subscribed (5%, CI = 2.12–4.76). This is expected, considering the fact that maternal health services are absorbed by the NHIS. Consequently, women who are subscribed to the scheme will be highly exposedto access the service as compared to their counterparts who are not subscribed to the scheme. Unlike the observation made among the poor, urban residents were less probable to utilise ANC as compared to rural women (−3%, CI = 0.98–1.64), however, married women in this category were more likely to utilise ANC than the unmarried (8%, CI = 0.74–2.86). Those residing in the Savannah zone were more probable to utilise ANC (2%, CI = 0.49–1.52) unlike their Forest zone counterparts (−3%, CI = 0.64–1.73) when compared with those in the Coastal zone (reference category).

Women in the middle wealth quintile who were obtaining water from sources other than pipe were relatively less probable to utilise ANC (−9%, CI = 0.65–2.71), however, those using modern contraceptives were more likely to utilise ANC (7%, CI = 0.95–2.63) as compared to those who were not. This has indicated that Ghanaian women using contraceptive also usually utilise ANC. As such, it can be inferred that women who are conscious about timing of their pregnancies are also much concerned about their pregnancies and unborn children. Those sharing toilet facility with other households (7.5%, CI = 0.56–2.61) together with those using public toilets (−5%, CI = 1.11–3.01) were less likely to access ANC as compared to women who were not sharing.

Urban rich women were relatively less probable to utilise ANC than rural rich women (−1%, CI = 0.28–2.73). This raises a number of concerns because it was expected that the rich who have wherewithal would make active use of all available health services. However, the emerging trend of wealthier women especially in the urban areas having the means of accessing other health care services they deem as ‘quality’ coupled with the general perception in the country that the NHIS is pro-poor could be adduced to the disinclination in usage stemming from the urban rich women. As compared to unmarried women, married women were more probable to access ANC (2%, CI = 0.75–3.16). Whilst low tendency of ANC utilisation was noted among those with primary education as compared with those without formal education (−3%, CI = 0.63–2.71), a contrary observation was made among women with at least secondary education (1%, CI = 0.63–2.71).

#### Logistic regression on place of delivery

Upon analysing the poor and place of delivery, poor women subscribed to the NHIS were 14% (CI = 1.42–2.13) more probable to deliver in health facilities than poor women who had not subscribed whilst poor urban residents also had a little higher propensity of delivering in health facilities (3%, CI = 1.66–3.21) over their rural counterparts (reference category) as indicated in Table 4. As compared to poor women who are affiliated to other religions, Christians were 9% (CI = 1.16–1.78) more likely to deliver in health facilities and a similar observation was made among married women as compared to unmarried ones (4%, CI = 1.08–1.71). Those listening to radio at least once a week had 10% (CI = 1.40–2.26) higher likelihood of delivering in health facilities than those who were not listening to radio at all.

Poor women resident in Savannah (9%, CI = 0.85–1.65) and Forest zones (5%, CI = 0.73–1.39) were more likely to deliver in health facilities just as working women when compared with non-working ones (5%, CI = 0.90–1.60). As shown in Table 4, poor women whose partners were engaging in secondary and tertiary occupation were more probable to deliver in health facilities (5%, CI = 0.86–1.60 and 7%, CI = 1.42–3.09 respectively). At the same time, poor women sharing toilet facilities with other households (1%, CI = 0.77–1.01) and those using public toilets (5%, CI = 0.98–1.23) were also more inclined towards health facility delivery than those who were not sharing toilet facilities (reference category).

Among women in the middle wealth status, those subscribed to the NHIS were 7% (CI = 1.00–2.10) more likely to deliver in health facilities over those who were not subscribed. Urban residents in the middle wealth status were 17% (CI = 1.63–3.44) more likely to deliver in health facilities than their rural counterparts whilst Christians were similarly more probable to deliver in health facilities than those affiliated to other religions (8%, CI = 0.75–1.80). With regard to educational status, women with primary (2%, CI = 0.98–1.15) and at least secondary education (9%, CI = 1.01–2.75) were more probable to deliver in health facilities than their counterparts without any formal education as indicated in Table 4. As compared with Coastal zone, women in the Savannah zone were less likely to deliver in health facilities (−2%, CI = 0.82–1.99) unlike Forest zone middle wealth status women (2%, CI = 2.14–3.11). Women who were working had 7% (CI = 1.78–2.56) higher likelihood of health facility delivery over non-working women.

Those who were not alone in terms of decision making about healthcare (−5%, CI = 0.63–1.21) and visits (−1%, CI = 0.69–2.11) were less probable to deliver in health facilities as compared to those taking those decisions alone (reference category). This points to the notion that in order for a woman be able to utilise health services, her decision making rights ought to be safe-guarded. However, women who were not deciding on household purchases alone were 9% more probable to deliver health facilities over those taking the decision alone. Again, as compared to those not sharing toilet facilities, women sharing with other households (4%, CI = 0.52–1.74) and those using public toilets (1%, CI = 1.55–3.02) were more inclined toward health facility delivery as shown in Table 4.

With regard to rich women, those subscribed to the NHIS were 5% (CI = 1.02–3.18) more likely to deliver in health facilities over those who were not subscribed and similar observation was made among rural residents (4%, CI = 1.60–5.92). At the same time, Christians were 3% (CI = 0.71–2.97) more probable to deliver in health facilities than other religious’ affiliates. Whilst those with at least secondary education were likely to deliver in health facilities (1%, CI = 1.12–2.84), rich women with primary education were less probable to deliver in health facilities as compared to their counterparts without any formal education (−4%, CI = 0.82–3.01) as shown in Table 4. Rich women whose partners had attained primary (4%, CI = 1.88–2.76) and at least secondary education (5%, CI = 2.71–3.98) were more likely to deliver in health facilities as compared to those whose partners had not been educated.

The study indicated that rich women who listened to radio at least once a week were 2% (CI = 2.71–4.87) more likely to deliver in health facilities over those who do not listen at all. Again, residents of Savannah (3%, CI = 1.53–3.44) and Forest zone (3%, CI = 2.31–5.44) more probable to deliver in health facilities than those in the Coastal zone. Working rich women were similarly more likely to deliver in health facilities than their non-working counterparts (2%, CI = 2.55–4.21) whilst those whose partners were engaged in secondary (7%, CI = 0.74–2.55) and tertiary occupation (7%, CI = 2.11–4.31) were more likely to deliver in health facilities over those whose partners were into primary occupation (reference category). Both rich women sharing toilet facility with other households (−2%, CI = 0.93–3.11) and using public toilet (−3%, CI = 1.71–3.85) were less likely to deliver in health facilities as compared with those who did not share as noted in Table 4. At 95% confidence interval, the three models fit reasonably well at probability values of 9.9%, 13.5% and 7.3% for the poor, middle and rich models respectively.

## Discussion

This study was motivated by reported evidence of variation in maternal healthcare utilisation across wealth status driven by diverse factors [20, 21]. It is evident from the study that utilisation of health service is a function of NHIS subscription as also revealed by an earlier study in Ghana [26]. From the descriptive results, majority of the poor subscribed to insurance compared to the middle and the rich, perhaps the reason for this can be the observed group of the emerging “intervened poor” in Ghana where NGOs are aiding the poor especially in the savannah zone of the country. It was evident from the study that NHIS subscription was low among women in the middle wealth status. Though unexpected, it is not surprising because to subscribe or otherwise is purely a choice or decision to be made and it was evident that among poor, middle and the rich independence in decision making on healthcare, household purchase and visit was least among women in the middle wealth status. Consequently, this might account for the observed poor NHIS subscription among women with middle wealth status.

The study has revealed that rich women are more inclined toward both ANC visits and health facility delivery than poor women. Rich women might be more likely to have easy financial access of any health services of their choice and as such can easily utilise maternal health services unlike their poor counterparts. Although ANC is free in Ghana, cost of transportation can be a barrier to dissuade poor women from regularly attending ANC thereby possibly accounting for the relatively low maternal healthcare utilisation among them despite the fact that no much variation persisted with regard to NHIS subscription. The inclination of high wealth status toward maternal healthcare utilisation buttresses some earlier findings [4, 24] that enabling factors motivating healthcare utilisation constitutes one’s wealth status just as also purported by the Behavioural Model [23].

The study has therefore pointed to the fact that in order for a woman to enjoy optimum access to maternal health services, she needs to be economically endowed. It is therefore not surprising for the high prevalence of maternal mortality to occur in developing countries where most women lack the economic capacity for accessing healthcare [1, 4]. Meanwhile obtaining better maternal healthcare assures desirable delivery outcomes which implies that as long as variation persist among the economically endowed and poor women, the latter will persistently fall prey to maternal induced complications.

Although poor women generally had lower utilisation of maternal health services, those subscribed to the NHIS were noted to have a relative higher access to ANC more than their unsubscribed counterparts. NHIS as cost cutting intervention has therefore proven be of extreme benefit in the quest to enhance maternal utilisation of healthcare especially among the poor. This indicates that NHIS serves as enabling factor irrespective of one’s wealth status as posited by the Behavioural Model (BM) of healthcare utilisation.

Urban poor women were noted to have relatively high tendency of ANC attendance than their rural counterparts. This is not surprising considering the fact that most health facilities in Ghana are clustered in urban settings. Again this observation may fit into the contextual predisposing factors postulated by the BM as comprising demographic and social composition of communities having the capacity to induce healthcare utilisation [23, 24]. Considering pattern of distribution of health facilities in Ghana, urban women generally have a relative advantage over rural women and as such their geographical access to health facilities may be easier as compared to rural residents thereby contributing to this observation.

Poor married women were more probable to access ANC. Women in marital unions are more likely to enjoy social support manifesting in either accompaniment to ANC by one’s partner or being reminded by the partner about ANC schedules. However, unmarried women will be lacking such social benefits and might thereby may be less motivated to access ANC especially when males who impregnated these women deny fathering the child. It has been similarly noted in Nepal that demographics such as being in a marital union is a source of social support that have higher inclination towards effective utilisation of health services [27].

Working poor women were having a higher tendency of utilising ANC as compared to non-working poor women. This finding may be as a result of relative higher exposure among working women arising from their frequent interaction with co-workers with whom they are more likely to discuss matter pertaining to their household arrangement and their health and wellbeing issues. As co-workers, they are likely to draw inspiration from each other; a social support that non-working women might lack. In this context, been employed or working will denote a motivating factor as inherent in the Behavioural Model.

Poor women whose partners had at least secondary education were noted to have higher ANC visits than those whose partners had no formal education. It is not surprising that such women have relatively high tendency to utilise ANC because education endows people to appreciate the essence of seeking healthcare at each stage of life and as such men with at least secondary education might urge their wives on the need to regularly access ANC without obstructing them. Juley-Anne [28] also found that educational attainment of both women and their partners is a major motivation for accessing health services and this was also earlier revealed [29].

Radio been one of the dominant mediums of mass media communication in Ghana was realised to have higher inclination toward ANC utilisation as poor women listening to radio at least once a week were 10% higher to utilise ANC than their counterparts who do not listen at all. This finding therefore highlights the instrumental role of mass media in shaping people’s lives as posited earlier [30].

The study revealed that poor women who could not decide independently for their own healthcare were less likely to access ANC as compared to those who could decide on their own. However, those who could decide on matters pertaining to household purchases and visits had higher propensities of ANC utilisation. This indicates how women’s autonomy feeds into critical spheres of their lives and therefore implies that autonomy of women is a prerequisite for enhancing their utilisation of healthcare services [31].

Among those in the middle wealth status category, on the contrary to what was found among poor women, urban residents were less probable to access ANC as compared with their rural counterparts. This might however originate from increasing job demands and overcrowding in urban settings as compared to rural areas. In one’s quest to make ends meet within urban settings, women are more likely to postpone ANC in order to meet job schedules and deadlines. Meanwhile those who might desire to regularly seek the ANC service might be discouraged by the crowd they would have to compete with.

The study has revealed that utilisation of ANC is facilitated within the domains of poor and middle wealth status women’s NHIS subscription, marriage, occupation as well as decision making autonomy with regard to owns’ healthcare, household purchases and visits. Again, usage of modern contraceptives manifested in high inclination toward ANC utilisation among women in the middle wealth status stratum as also noted among poor folks.

Among rich women, although it was realised that those who had subscribed to the NHIS were highly inclined toward ANC, it was less as compared to subscribed poor women with high ANC utilisation. This indicates that interventions geared toward enhancement of maternal healthcare utilisation and combating adverse occurrences aligned with maternity must focus on poor women. This is because literature have continuously highlighted less utilisation of healthcare among poor women and even when they use, they usually access substandard care due to the element of cost [26, 32].

With regard to place of delivery, most factors that favoured ANC utilisation were noted to also favour health facility delivery across all wealth quintiles. This is an indication that motivating factors aligned with a specific maternal service utilisation might be a universal facilitator as far as maternal healthcare access is concerned. On the contrary, it implies that factors obstructing a specific maternal healthcare service use may obstruct utilisation of all maternal healthcare services.

Specifically, subscription to NHIS, marriage and educational attainment were all contributing factors towards health facility delivery among poor women. It can therefore be inferred that a woman’s ability to deliver in healthcare facility is hinged on numerous factors [33–35]. It was also noted that poor women residing in savannah and forest zones were more probable to deliver in health facilities. This might imply that enabling factors available to these women abound as purported by Anderson’s Behavioural Model. Since women usually desire better health conditions for themselves and their children alike, they are more likely to utilise health facility for delivery purposes whenever an enabling environment avails itself [33] and as such manifesting in high utilisation of health facilities for delivery.

With rich women in context, they exhibited similar traits as their colleagues in the poor and middle wealth categories. This indicates that to a greater extent, obstructing and enabling factors surrounding maternal healthcare are near universal except few domains. However, the study has illustrated that as compared with rich women, cost saving interventions are much beneficial to the poor.

### Limitations of the study

It is worthy to acknowledge that the study has some limitations. The study made use of cross sectional data and as such could not explore cause and effects of the findings observed. Also, the study was unable to investigate the motivation that urged women to attend ANC and also utilise health facilities for delivery due to the secondary data used. In spite of these, limitations the results present a credible reflection on the interaction between maternal healthcare utilisation and NHIS subscription and further offer practical measures that would aid to further improve maternal healthcare utilisation in the country.

## Conclusions

With the aid of the Anderson’s Behavioural Model (BM) of healthcare utilisation, this study has drawn attention to some key interventions worth instituting to accelerate maternal healthcare utilisation in the country. For instance, it has demonstrated the need for well-tailored maternal healthcare interventions such as offering motivation in terms of providing gifts to women who regularly attend ANC and those utilising health facilities for delivery. This is likely to boost maternal healthcare utilisation in the country and such interventions must target the poor and less privileged in society in order to further enhance maternal healthcare utilisation. It can be achieved through collaboration between the Ghana Health Service and other donor agencies committed to maternal and child health in the country such as Savanna Signatures and Care Net Ghana. There is also the need for the Reproductive and Child Health (RCH) unit of the Ghana Health Service to intensify mass media campaigns projecting the essence of ANC and delivery in health facilities since mass media (specifically radio and television) have proven to have higher inclination towards maternal healthcare utilisation. Again, female education must be targeted and promoted in order for them to ascend on the academic ladder since the study has revealed that highly educated women utilise maternal health services; that is having high tendency of attending ANC and utilising health facilities for delivery more than their uneducated counterparts.
